# Evaluation of Osseous Integration of PVD-Silver-Coated Hip Prostheses in a Canine Model

**DOI:** 10.1155/2015/292406

**Published:** 2015-01-28

**Authors:** Gregor Hauschild, Jendrik Hardes, Georg Gosheger, Sandra Stoeppeler, Helmut Ahrens, Franziska Blaske, Christoph Wehe, Uwe Karst, Steffen Höll

**Affiliations:** ^1^Department of Orthopedics and Tumor Orthopedics, University Hospital of Muenster, Albert-Schweitzer-Campus 1, 48149 Muenster, Germany; ^2^Department of General and Visceral Surgery, University Hospital of Muenster, Albert-Schweitzer-Campus 1, 48149 Muenster, Germany; ^3^Institute of Inorganic and Analytical Chemistry, University of Muenster, Corrensstraße 28/30, 48149 Muenster, Germany

## Abstract

Infection associated with biomaterials used for orthopedic prostheses remains a serious complication in orthopedics, especially tumor surgery. Silver-coating of orthopedic (mega)prostheses proved its efficiency in reducing infections but has been limited to surface areas exposed to soft tissues due to concerns of silver inhibiting osseous integration of cementless stems. To close this gap in the bactericidal capacity of silver-coated orthopedic prostheses extension of the silver-coating on surface areas intended for osseous integration seems to be inevitable. Our study reports about a PVD- (physical-vapor-deposition-) silver-coated cementless stem in a canine model for the first time and showed osseous integration of a silver-coated titanium surface *in vivo*. Radiological, histological, and biomechanical analysis revealed a stable osseous integration of four of nine stems implanted. Silver trace elemental concentrations in serum did not exceed 1.82 parts per billion (ppb) and can be considered as nontoxic. Changes in liver and kidney functions associated with the silver-coating could be excluded by blood chemistry analysis. This was in accordance with very limited metal displacement from coated surfaces observed by laser ablation inductively coupled plasma-mass spectrometry (LA-ICP-MS) 12 months after implantation. In conclusion our results represent a step towards complete bactericidal silver-coating of orthopedic prostheses.

## 1. Introduction

Due to recent advances in endoprosthetic design that have led to lower complication rates, particularly with regard to mechanical failure and aseptic loosening [1–3], megaprostheses for limb salvage procedures have increasingly gained acceptance [1]. Nevertheless periprosthetic infection of megaprostheses continues to be a common and serious complication in orthopedic oncology [4, 5]. The consequences of this specific complication can be disastrous resulting in ultimately secondary amputation in some cases [6, 7]. Therefore, reducing periprosthetic infection rates at present is one of the most important topics dealing with limb salvage procedures. As far as eradication of biofilm-producing bacteria from the prosthesis' surface in most cases is not possible [8], prophylactic prohibition of bacterial ingrowth using surface modifications by silver-coating has recently proven to be an effective approach [6, 9, 10]. In a previous study, our group demonstrated high antimicrobial activity in conjunction with the lack of toxicological side effects [6]. However, one weakness of this approach was that the body of the prosthesis was silver-coated only because of concerns regarding a possible inhibition of osseointegration with the use of cementless silver-coated stems. Indeed, we could demonstrate that silver ions do not inhibit osteoblasts in low concentrations* in vitro* compared to titanium, but it is well known that silver exhibits dose-dependent cytotoxic effects on mammalian cells [11]. To the best of our knowledge, there are no studies about the osseointegrative properties of cementless silver-coated stems* in vivo* at present. Therefore, this study investigates the osseointegration of silver-coated stems in hip prostheses in a canine model in order to create a homogeneous silver-coating of both the stems and the prosthetic body.

## 2. Methods

Animal experiments were conducted under an ethics committee approved protocol in accordance with German federal animal welfare legislation (Az 33.9-42502-04-07/1362), which is in compliance with the guidelines outlined in the NRC Guide for the Care and Use of Laboratory Animals. All animals were housed in groups of two (*N* = 3) and three (*N* = 1) at the Laboratory Animal Science of the Hannover Medical School, Germany. All procedures associated with the experimental animals were performed in this same facility.

### 2.1. Study Design

The study followed a prospective randomized end-point design. Nine healthy female beagle dogs (Harlan Laboratories UK LTD, UK) with an average body weight of 10.3 kg and a mean age of 2.2 years were included. After randomized decision for side of implantation (left or right hip joint) all animals underwent unilateral total hip replacement using a custom made physical-vapor-deposition- (PVD-) silver-coated titanium alloy stem (Implantcast GmbH, Buxtehude, Germany) in addition to a cemented polyurethane cup (Biomedtrix, Boonton, USA) and a modular head (Implantcast) each. Follow-up was about 12 months including clinical assessment, blood count, blood chemistry, C-reactive protein, metal ions, and X-rays as scheduled in Table 1. After death biomechanical testing as well as histological examinations and laser ablation inductively coupled plasma-mass spectrometry (LA-ICP-MS) of the prosthesis-bone-interface were performed with randomized assignment of the femora.

### 2.2. Stem

The custom-made noncemented stem was developed by Implantcast (Buxtehude, Germany) in accordance with the Clinic for Orthopedics and Tumor Orthopedics, University Hospital Münster (Westfälische Wilhelms-Universität Münster, Germany), and consisted of wrought titanium aluminum-6 vanadium-4 alloys (DIN ISO 5832-3:2000–08). Its hexagonal appearance with trimmed edges in site of Adams' bow significantly differs from all available stem configurations in veterinary medicine and is intended to provide rotational and a high primary stability.

The sandblasted surface with a topographical depth of 50 *µ*m should allow easy osseous integration of the prosthesis. Nine sizes (−1 to 7) were manufactured and designed for diaphyseal press fit implantation. Two of them (−1 and 0) have been used for implantation in this study. The modular connection surface and modular head were in accordance with the design of the commercially available cup components by Biomedtrix (USA).

### 2.3. Silver-Coating

**Figure 1:**
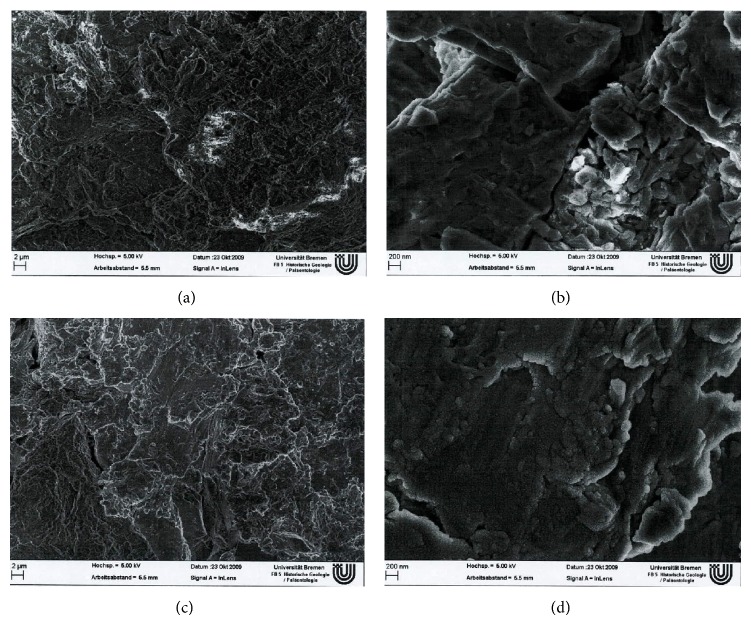
SEM of titanium discs: upper row shows uncoated titanium disc topography in micro- (left) and nanoscale (right); row below shows silver-coated titanium disc topography with no alterations in the microscale but a slightly smoothend appearance of the surface contour without perceptible changes in topography in the nanoscale.

Ag/SiO_*x*_C_*y*_ plasma-polymer-coating of the stem (Bio-Gate AG, Bremen, Germany) was performed as described by Khalilpour et al. [13]. Elemental silver particles were sputtered onto the surface of the stem in a physical-vapor-deposition (PVD) process and covered by a SiO_*x*_C_*y*_ plasma polymer layer using a subsequent chemical vapor deposition (CVD) process with hexamethyldisiloxane as precursor. According to the manufacturer coating was completed including overall 9 layers (4 Ag layers, 5 SiO_*x*_C_*y*_ layers; thickness of one complete layer Ag + SiO_*x*_C_*y*_: 30–50 nm) resulting in a mean Ag content of 8.1 *µ*g/cm^2^ and a maximum coating thickness of up to 300 nm. The total silver content for the stem sizes used in this study was calculated as 99 *µ*g for size −1 and 179 *µ*g for size 0.  Figure 1 shows SEM scans of uncoated and coated titanium discs in comparison.

### 2.4. Surgical Procedure

All surgical procedures were performed within the operating theater of the Laboratory Animal Science of the Hannover Medical School.

After intravenous induction of general anaesthesia, radiographs of both hip joints in ventrodorsal as well as laterolateral direction were conducted for preoperative planning. Following aseptic preparation of the operation field, presurgical application of a standard antibiotic (penicillin-G procaine 10,000 I.E./kg body weight/dihydrostreptomycin, Intervet, Germany) as well as a NSAID (carprofen 4 mg/kg body weight, Pfizer, Germany) and placement of the animal in lateral recumbency, a standard craniolateral approach was made to expose the hip joint. Femoral head and neck resection was followed by cemented implantation (Refobacin-Palacos, Heraeus, Germany) of a standard polyethylene cup (Biomedtrix, Boonton, USA). Preparation of the femur was performed using size-adapted (0.5 mm undersized) femoral broaches (Implantcast GmbH, Germany) for press fit implantation of a custom-made femoral stem (Implantcast GmbH, Germany) and apposition of a modular head. Repositioning of the hip joint as well as capsula and wound closure followed standard procedures.

### 2.5. Postoperative Treatment

Immediately postoperative radiographs were conducted following the similar positioning procedure described above for presurgical imaging. For postsurgical analgesia, a standard NSAID (carprofen) was administered for additional 14 days accompanied by buprenorphine on demand. Anti-infective therapy was continued as started over a 10-day period. All animals underwent restricted ambulation for a period of 14 days before being housed in groups.

### 2.6. Blood Count, Blood Chemistry, and C-Reactive Protein

Blood count and blood chemistry (ALT/GPT, GLDH, AST, ALP, bilirubin, UREA, creatinine, phosphate [anorg.], cholesterol, GLU, total protein, ALB, and electrolytes) evaluation was performed at the central laboratory of the Small Animal Clinic of University of Veterinary Medicine, Foundation, Hannover, Germany. C-reactive protein was analyzed by Biocontrol, Ingelheim, Germany.

### 2.7. Ag Quantification in Serum

Silver trace elemental concentrations in the serum samples, collected as scheduled in Table 1, were quantified by means of inductively coupled plasma-mass spectrometry (ICP-MS). In detail, the samples were diluted 50-fold with highly purified nitric acid in bidistilled water (0.2% (v/v), Merck KGaA, Darmstadt, Germany). Additionally, they were spiked with indium (Merck KGaA) as matrix effect monitoring internal standard to a final concentration of 1 *µ*g L^−1^ prior to direct, autosampler-assisted (model SC4-S, Elemental Scientific Inc., Omaha, NE, USA) aspiration into the utilized ICP-MS (model iCAP Qcm, Thermo Fisher Scientific Bremen GmbH, Bremen, Germany). This quadrupole-based ICP-MS system was equipped with a dedicated collision/reaction cell (QCell), which was pressurized at 4.68 mL min^−1^ with a mixture of 6% hydrogen in helium (purity: 99.999%) in order to remove spectral, polyatomic interferences like ^91^Zr^16^O^+^ by means of kinetic energy discrimination (3 V bias between QCell and quadrupole).

Both silver isotopes, namely, 107 and 109, were investigated with a quadrupole dwell time of 0.02 s in 8 independent runs. All samples were analyzed in triplicate and the mean values were used for subsequent concentration calculations against an external calibration ranging from 0 to 15,000 ng L^−1^. Further instrument parameters were as follows: nebulizer = PFA *µ*Flow ST (Elemental Scientific Inc.); spray chamber: quartz glass, cyclonical shape with baffle, cooled to 2.7°C; sample pumping speed = 300 *µ*L min^−1^ using an external peristaltic pump equipped with flared-end polyvinylchloride tubing, ID = 0.508 mm; power = 1,550 W, cool gas flow = 14.0 L min^−1^, auxiliary gas flow = 0.9 L min^−1^; nebulizer gas flow = 0.91 L min^−1^; sampling depth = 5 mm; 2nd extraction lens voltage = −133.9 V; injector pipe inner diameter = 1.8 mm; sampler material = Ni; skimmer material = Ni with 2.8 mm linear insert. According to the 3*σ* criterion, the limit of detection (LOD) was estimated to be 0.9 ng L^−1^ with a sensitivity of 45.4 and 44.4 cps per *µ*g L^−1^ in the diluted matrix, respectively. The squared correlation coefficient resulted in 0.999, while matrix spikes revealed an averaged recovery of 102.3%.

### 2.8. Radiological Evaluation

Estimation of modes of failure of the femoral part of the total hip prosthesis had been performed in accordance with the Gruen method [14]. Evaluation was carried out using nondigitized conventional full-size radiographs in the scale 1 to 1. Signs of osteolysis ≤ 1 mm were estimated as not loose, and signs of osteolysis with dimension of 1-2 mm without progress after six to 12 months likewise were evaluated as not loose. Loosening was identified with a dimension of osteolysis of ≥2 mm and progress [15].

### 2.9. Biomechanical Testing

**Figure 2:**
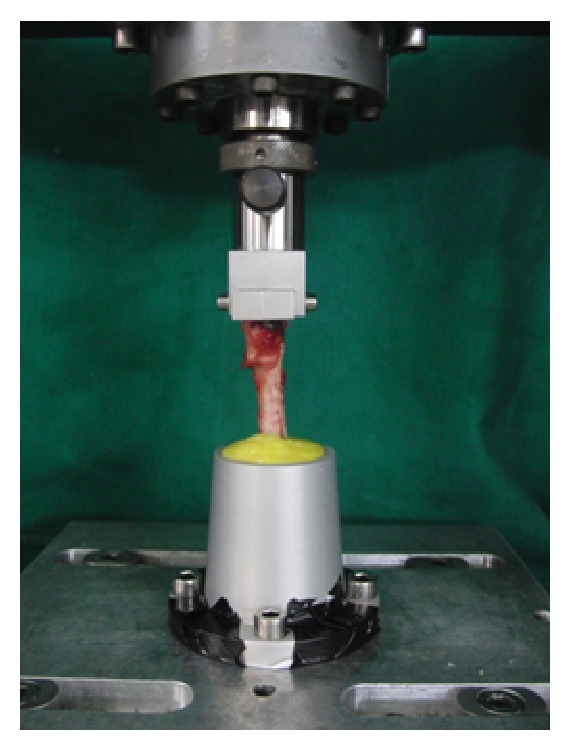
Femur fixed in the biomechanical testing system.

Pull-out tests were conducted using a biomechanical test system (Z005 Zwick/Roell, Ulm, Germany) measuring the vertical pulling force to failure in Newton (N). Prior to biomechanical testing the femora were frozen at −70°C and thawed 1 hour before starting the pull-out procedure. To ensure perpendicular force to the implant a custom-made adaptor connected the neck of the implant placed* in situ* with the test system while the distal part of the femur was fixed in polymethylmethacrylate (PMMA, Technovit 3040 Fa. Heraeus, Hanau, Germany) (Figure 2). Maximum pull-out force in Newton was recorded by Test Expert Software (Version 10.11 Zwick/Roell, Ulm, Germany) and Microsoft Excel 2007 to map secondary stability data representing osseous integration. Additional cadaver testing data of an earlier study [12] including four uncoated implanted femora representing primary stability was considered to record baseline data.

### 2.10. Histological Evaluation

Immediately following death the randomized assigned femora with implant* in situ* were referred to preparation for undecalcified histological evaluation. Nine regions of interest (ROI) had been determined for each stem size implanted resulting in nine transsectional cutting areas perpendicular to the longitudinal axis of the femur. Starting measurement at the shoulder of the prosthesis, the cutting areas had been located for stem size −1 at 3, 8.75, 14.5, 20.25, 26, 31.75, 37.5, 43.25, and 49 mm and for stem size 0 at 3, 9.75, 16.5, 23.25, 30, 36.75, 43.5, 50.25, and 57 mm in distal direction. The histological sections were prepared using a modified method according to Donath [16, 17] and Hahn et al. [18]. Here, a cutting-grinding technique for metal containing tissue not suitable to be sectioned by routine methods was applied. First, the histological specimens were fixed with formaldehyde solution, followed by dehydration. After infiltration with a 1 : 1 mixture of glycol methacrylate and Technovit 7200 VLC (Heraeus Kulzer, Wehrheim, Germany), the embedding in pure Technovit 7200 VLC was carried out. Polymerization was accomplished using an* Exakt*-light-polymerization unit (Exakt Technologies, Oklahoma City, OK, USA) at wavelength of 400–500 nm. The embedded tissue specimens were sectioned (200 *µ*m) using an* Exakt*-cutting-grinding system with a diamond-impregnated saw blade (Exakt Technologies). To reduce the thickness, all samples were grinded down using diamond-coated plates. The prepared cross sections were of a final thickness between 10 and 17 *µ*m. Two slices were prepared for each ROI with a distance of 600 *µ*m. The first of these then was stained with toluidine blue for histological evaluation carried out by PaTH, Hannover, Germany. In addition to qualitative evaluation, percentage of osseous ingrowth onto the stem surface had been focused on. The second slice was saved unstained for the LA-ICP-MS analysis.

### 2.11. Laser Ablation Inductively Coupled Plasma-Mass Spectrometry (LA-ICP-MS)

Highly sensitive analysis of the bone-implant-interface was performed using a laser ablation system (LSX 213, CETAC Technologies, Omaha, NE) coupled to an inductively coupled plasma quadrupole mass spectrometer (iCAP Qc, Thermo Fisher Scientific, Bremen, Germany) as described by Blaske et al. [19] to address possible migration of metal components from the stem surface into the surrounding tissue. A collision/reaction cell was used for quadrupole-based MS detection to discriminate polyatomic ions (^91^Zr^16^O^+^), resulting from the blasting process of the titanium stem, which could possibly interfere with ^107^Ag^+^, by their kinetic energy.

### 2.12. Statistics

A percentage of osseous ingrowth determined from the histological analyzed ROIs was paired and matched to the corresponding Gruen zones analyzed by the radiological evaluation.

## 3. Results

### 3.1. Drop-Out

One dog had to be removed from the study due to complications not associated with the surgical implant (V5). Overall, nine from 10 dogs underwent the analytical procedures as given in Table 2.

### 3.2. Hematological Evaluation

No systemic side effects associated with the implant or surgical process had been detected. During the follow-up period blood count and chemistry values were, in accordance with current reference ranges [20], not other than normal. Values of C-reactive protein showed a slight increase on day 3 after surgery followed by a decrease nearly up to normal until day 10 after implantation (Table 3).

### 3.3. Silver Concentrations in Serum

Mean values of silver trace elemental concentrations in the collected serum samples ranged from 0.20 ppb (±0.01 ppb standard deviation) to 1.82 ppb (±0.01 ppb standard deviation). Baseline data representing normal values ranged from 0.20 to 1.38 ppb.

### 3.4. Radiological Evaluation

Due to failed implantation in one case (V1; stem perforating the diaphysis, Gruen-zones only evaluable in parts), eight test animals could be included for radiological evaluation. One of them (V7) underwent revision surgery of the stem. According to the criteria of Gruen as well as Engh, four of eight analyzable stems implanted (50%) were classified as “stable” ( V2, V3, V9, and V10) while another four (50%) were showed to be “loose” (V4, V6, V7, and V8).

### 3.5. Biomechanical Testing: Pull-Out Tests

The maximum pull-out force (*F*_max⁡_) of implanted cadaver femora representing primary stability [12] ranged from 68.42 N to 625.13 N with a mean of 225.95 N (±266.84 N standard deviation). Five of nine implanted femora (V1, V3, V4, V8, and V10) were randomly assigned for biomechanical testing. For these PVD-silver-coated titanium stems *F*_max⁡_ ranged from 0 to 2008.6 N with a mean of 439.62 N (±864 N standard deviation) (Table 4). Based on this data, two of five were classified as stable (V3, V10).

### 3.6. Histological Evaluation

Four of nine implanted femora were randomly assigned to histological evaluation (V2, V6, V7, and V9). Percentage of osseous integration of the silver-coated stem versus connective tissue membrane surrounding the implant ranged from 0 to 100% (Table 5). Implant areas localized outside the femur have been excluded from analysis. To match histological data with the X-ray analysis, ROIs were allocated to the respective Gruen zones (Table 6). For histologically analyzed stems classified radiologically as stable (V2, V9) a mean percentage of osseous integration was 63.6% (±31.5%) while stems radiologically classified as loose (V6, V7) showed a percentage of osseous integration of 5.6% (±6.8%). Overall two of four histologically analyzed stems were classified as stable (V2, V9).

### 3.7. Visualization of Metal Components' Displacement Laser Ablation Inductively Coupled Plasma-Mass Spectrometry (LA-ICP-MS)

**Figure 3:**
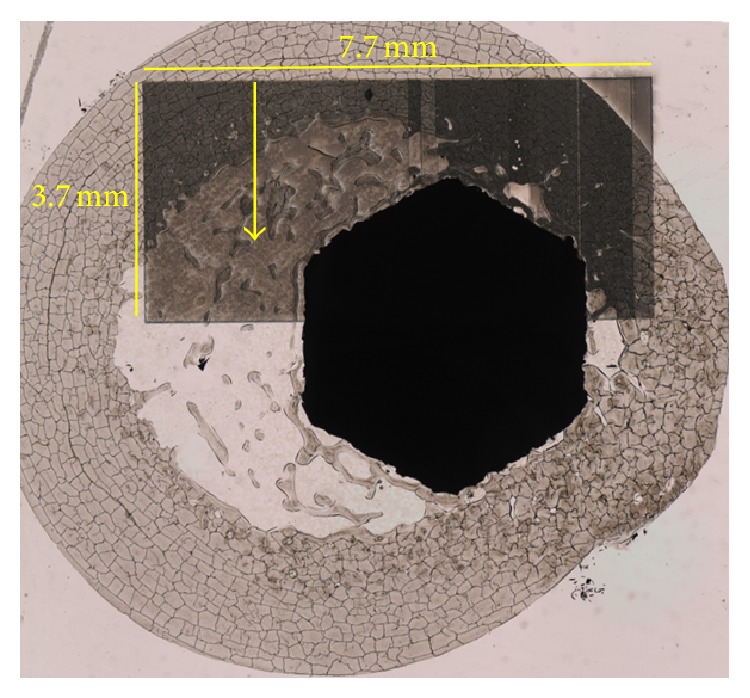
Photomicrograph of ROI 8 (V2) showing area of ablation and scan direction (arrow).

**Figure 4:**
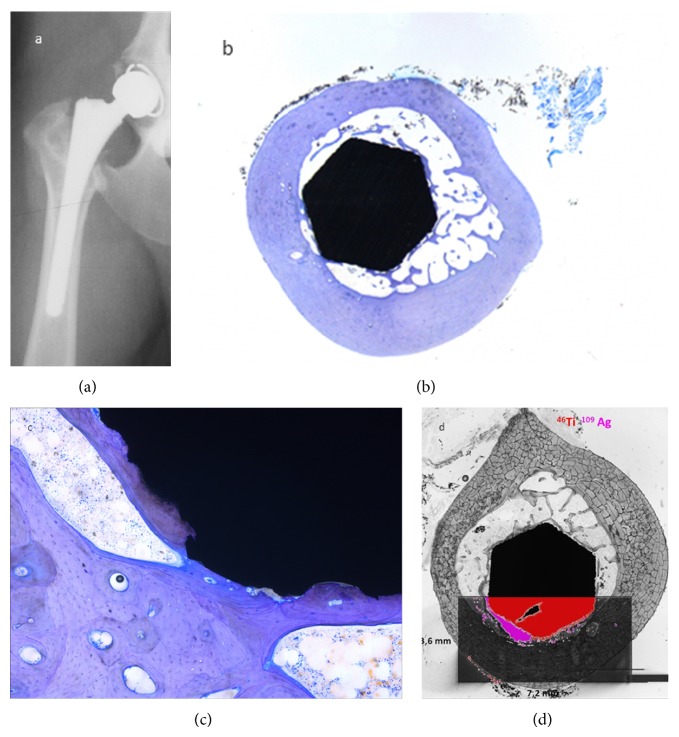
V9 ROI 6, 12 months after surgery: X-ray in v/d recumbency showing (a) stable osseous integration of the stem, (b) histological analysis in overview and detail of ROI 6, and toluidine blue staining. 10x magnification: (b, c) osseous ingrowth on 75% of the stem circumference and (d) LA-ICP-MS scan of ROI 6 displaying the overlay images of titanium (Ti) and silver (Ag).

**Figure 5:**
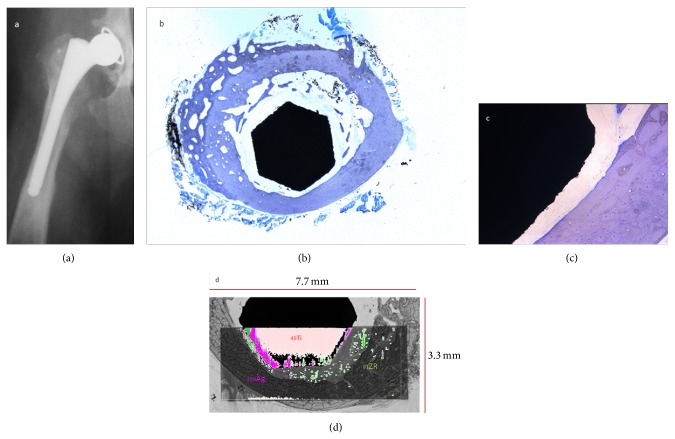
V7 ROI 8, 12 months after surgery: X-ray in v/d recumbency showing (a) loosening of the stem, (b) histological analysis in overview and detail of ROI 8, and toluidine blue staining. 10x magnification: (b, c) osseous ingrowth on 0% of the stem circumference and (d) LA-ICP-MS scan of ROI 6 displaying the overlay images of titanium (Ti) and silver (Ag).

Four femora (V2, V6, V7, and V9) assigned to histological evaluation also were referred to LA-ICP-MS as described above. Considering the complex sample structure as well as technical determinations inclusion of the implant, connective tissue/soft tissue areas, cancellous bone, and cortical bone into one ablation area (Figure 3) required a preselection of slices representing the ROIs as follows: ROIs 6, 8, and 9 were scanned for V2, ROIs 6, 7, and 8 for V6, ROIs 6 and 8 for V7, and ROIs 6 and 9 for V9. Metal displacement from the implant surface into surrounding tissues could be observed for all samples regarding silver, titanium, and zirconium without true patterns of distribution becoming obvious. Cancellous bone in the implant-host interface showed a diffuse allocation of silver signals whereas connective tissue membranes surrounding the stem exhibited minor but revealed a cumulation of silver signals located at the soft tissue-cortical bone interface. Only isolated spots of silver signals could be determined in cortical bone. This was also true for different regions of the stem in each ROI analyzed. Zirconium distribution followed similar allocations but compared to silver, more signals were detected at the implant surface whereas only very small amounts of titanium could be detected in displacement from the stem surface (Figures 4 and 5).

## 4. Discussion

The concept of prophylactic silver-coating of orthopedic implants is based on the long-time known and proven antimicrobial effect of silver in medical use [21]. Despite its potential for toxicity to mammalian cells [22], the bactericidal activity of silver at concentrations as low as 35 ppb [23, 24] in conjunction with a threshold for clinically apparent cell toxicity of 300 ppb [23, 25–27] enables the application of this concept even in case of extended surface areas as given in orthopedic megaprostheses. Previous papers suggested the anti-infective efficiency of this approach without associated side effects in experimental as well as clinical trials [6, 8, 9, 11]. Since silver-coating at present has been limited to surface areas apposed to soft tissue not including the articulating surface and especially the stem which is intended for osseous ingrowth in this study, we aimed to transfer this approach to the stem to close an existing gap in bactericidal capacity of silver-coated prostheses.

Focusing osseous ingrowth onto silver-coated titanium surfaces, the canine model used here is well accepted and is seen as superior experimental model due to the secondary structure of osteons similar to the anatomic environment in humans [28, 29]. The titanium alloy appearance of the stem is in accordance with the current standard design of cementless total hip prostheses. Getting in contact with biologically active tissue titanium induces the release of cytokines, proteolytic enzymes, superoxides, and hydrogen peroxide generating a hydrated titanium-peroxide-matrix which enables a better osseous ingrowth compared with other alloys [30–32]. Surface topography at the micrometer level is to be seen as essential for bone response as well but standards are not established yet [32]. In a previous study, an uncoated finish of the custom-made titanium stem was used in a canine model showing median maximum pull-out forces *F*_max⁡_ of 3764 N with 1515.35 N standard deviation [12] indicating secondary stable osseous integration of the prostheses and confirming the suitability of the created stem finish for uncemented implantation. In the present trial, four of nine implanted PVD-silver-coated titanium stems were classified as stable proving the suitability of the coating concept for osseous ingrowth in principle. Maximum pull-out forces of two of five biomechanically tested femora (414.55 N, 2008.83 N) exceeded mean values measured for primary stability as well as for cemented implantation [12] but did not reach mean secondary stability values of uncoated titanium stems. Due to the fact that stems classified as stable in radiological evaluation proved to be stable in histological as well as biomechanical estimation, a similar range of values of maximum pull-out force may be expected for two further femora not biomechanically but histologically tested.

The reason for this reduced secondary stability of the implant-host interface remains unclear. Aspects affecting the biomechanical strength of the osseous ingrowth leading to reduced secondary stability or loosening of the stem may be seen in alterations of the surface topography by the silver-coating process, cytotoxic effects of the silver-coating, and biochemical processes of the coating components.

Adhesion of osteoblasts demands a surface roughness ranging from 0.1 to 10 *µ*m [33–36]. Since Ewald et al. [37] described similar surface structures and only marginal increase in roughness from 2.8 to 4.5 *µ*m after deposition of a 2 *µ*m layer of silver using a PVD technique, the maximum coating thickness of 300 nm provided by the PVD-silver-coating process used in this study is not intended to significantly alter the topographical characteristics of the stem. Electron microscope scans (SEM) in the microscale (5k× magnification) revealed no differences in the isotropic surface topography between both finishes while in the nanoscale (50k× magnification) the surface showed a minimally smoothed appearance leaving the basic topographical structure unchanged (Figure 1).

Cytotoxic effects of the PVD silver-coating used in this study have been ruled out by indirect and direct contact assays according to the standard test method ISO 10993-5 of medical devices showing no reduction in mitochondrial activity, viability, and cell integrity of L-929 mouse fibroblast cells after 24 h of incubation or contact, respectively [13]. Previous* in vitro* trials of our group confirmed the dose-dependent effect of silver on osteosarcoma cells (HOS-58 SAOS-2). Adding up to 10 mg silver powder onto 20,000 cells starting density revealed an increase in alkaline phosphatase activity compared to control indicating a stimulation of osteoblastic differentiation. In contrast to that, adding 15 to 25 mg silver powder showed a marked decrease in marker activity compared to titanium alloy control [11]. Considering the total silver content of 99 *µ*g (stem size: −1) and 179 *µ*g (stem size: 0) of the implants used in the current study, these values are much below the threshold of osteoblastic cell toxicity stated in the* in vitro* trail. Very low silver trace elemental concentrations in the serum samples, at less than one percent of the threshold of 300 ppb, as well as the very low tendency of displacement of surface components underlined the health safety of the Ag/SiO_*x*_C_*y*_ plasma-polymer-coating. If biochemical processes caused by the silver-coating after implantation may impair the titanium-induced synthesis of bone matrix and thereby comprise osseous ingrowth is not expected but nevertheless subject to further investigation.

Despite lacking a true pattern of distribution, the degradation process of the silver-coating analyzed by LA-ICP-MS could, to the same extent, be detected for stems classified as stable or loose, respectively. Therefore, it appears unlikely that degradation of the coated surface over a one-year period impairs the osseous ingrowth. However, due to the small population of animals used in this study, further investigations are inevitable to confirm this hypothesis.

In the light of these considerations, we do not assume the PVD-silver-coating to significantly impair the stability of the implant-bone interface in a way that it will become clinically apparent despite impairments by the sum of marginal topographical, cytotoxical, and biochemical alterations of the surface compared to uncoated titanium alloy finishes causing reduced secondary stability that cannot be ruled out. It is more likely that inadequate stem sizes caused loosening and even may have compromised the biochemical strength of the bone-stem interface in the samples classified as stable. In this study, we had unexpectedly small sizes and low weights of our test animals making construction and production of new (smaller) stem sizes necessary. These new sizes did not fit the medullary cavity in an ideal way leading to reduced primary bone-implant contact. In case of an inadequate contact area, extended micromovement will cause fibrous connective tissue ingrowth. The fibrotic appearance of this tissue inhibits further bone-implant contact and leads to enhanced movement and loosening of the prosthesis [38, 39]. Due to the small population of test animals, which is to be seen as a major shortcoming of this trial, these results have to be reported as a proof of principle showing a tendency of osseous tissue for stable integration with a silver-coated titanium stem. Further experimental studies have to be carried out to confirm these preliminary findings.

## 5. Conclusion

In this study, we have shown that four of nine PVD-silver-coated titanium stems underwent stable osseous integration after implantation in a canine model. Local and/or systemic toxic side effects did not occur, which confirmed previous experimental and clinical work in the field.

## Figures and Tables

**Table 1:** Schedule of analytical procedures *in vivo*.

Procedures									
Clinical assessment	o		o	o	o	o	o	o	o
Blood count	o		o	o	o	o	o	o	o
Blood chemistry	o		o	o	o	o	o	o	o
C-reactive protein	o		o	o	o	o	o	o	o
Metal ions (serum analysis)	o		o	o	o	o	o	o	o
X-rays	o	o			o	o	o	o	o

	Pre-op	Post-op	3 days post-op	10 days post-op	6 weeks post-op	3 months post-op	6 months post-op	9 months post-op	12 months post-op

**Table 2:** Assignment of test animals to the analytical procedures and classification.

ID	Blood count/chemistry	Silver quantification	X-rays	Classification	Biomechanical testing	Classification	Histology	Classification	LA-ICP-MS
V1^x^	✓	✓	n.a.	—	✓	Loose	—	—	—
V2	✓	✓	✓	Stable	—	—	✓	Stable	✓
V3	✓	✓	✓	Stable	✓	Stable	—	—	—
V4	✓	✓	✓	Loose	✓	Loose	—	—	—
V6	✓	✓	✓	Loose	—	—	✓	Loose	✓
V7^y^	✓	✓	✓	Loose	—	—	✓	Loose	✓
V8	✓	✓	✓	Loose	✓	Loose	—	—	—
V9	✓	✓	✓	Stable	—	—	✓	Stable	✓
V10	✓	✓	✓	Stable	✓	Stable	—	—	—

n.a.: not analyzable; ^x^failed implantation; ^y^failed implantation followed by revision surgery.

**Table 3:** Presurgical and follow-up values of C-reactive protein (mg/L, normal value <5).

	Pre-op	3rd day post-op	10th day post-op	6 weeks post-op	3 months post-op	6 months post-op	9 months post-op	12 months post-op
V1	7.5	23.6	10.5	<5	<5	<5	8.5	<5
V2	<5	15.2	<5	<5	6.1	<5	<5	<5
V3	5.6	38.7	14.9	<5	<5	<5	<5	8.8
V4	<5	27.8	<5	<5	<5	5.8	<5	<5
V6	<5	35	13.9	<5	<5	<5	<5	<5
V7	<5	36.7	11	<5	<5	<5	<5	<5
V8	<5	26	9.6	<5	<5	<5	<5	<5
V9	<5	32.6	12.3	<5	<5	<5	<5	<5
V10	<5	37.5	9.6	<5	<5	5.9	<5	<5

**Table 4:** Maximal pull-out forces.

	*F*_max⁡_ (N)	Classification
C1^‡^	625.13	—
C2^‡^	93.75	—
C3^‡^	116.5	—
C4^‡^	68.42	—
V1	20.02	loose
V3	414.55	stable
V4	25	loose
V8	0	loose
V10	2008.63	stable

C1–4: cadaver implantation, ^‡^data by Welz 2008 [12]; V1–V10: test animals implanted.

**Table 5:** Percentage of osseous integration.

ROI	V2		V6		V7		V9	
	OI/%	CTI/%	OI/%	CTI/%	OI/%	CTI/%	OI/%	CTI/%
1	n.a.	n.a.	20	80	n.a.	n.a.	n.a.	n.a.
2	n.a.	n.a.	15	85	n.a.	n.a.	n.a.	n.a.
3	5	95	5	95	n.a.	n.a.	60	40
4	0	100	3	97	10	90	85	15
5	40	60	5	95	15	85	75	25
6	45	55	0	100	0	100	75	25
7	65	35	0	100	5	95	70	30
8	100	0	0	100	0	100	75	25
9	100	0	0	100	0	100	95	5

Mean	51 ± 41	49	5 ± 7	95	5 ± 6	95	76 ± 11	24

OI%: percentage of osseous integration of the stem; CTI%: percentage of connective tissue ingrowth between stem and surrounding tissue; n.a.: not analyzable, extraosseous localization of the stem areas.

**Table 6:** Matching of histological ROIs and Gruen zones.

ROI	Gruen zone	V2		V6		V7		V9	
		%	mm	%	mm	%	mm	%	mm
1	1	n.a.	0	20	1	n.a.	1	n.a.	0
4, 5, 6	2	28.3	0	2.7	1	8.3	1	78.3	0
7, 8, 9	3	88.3	0	0	1	1.7	1	80	0
7, 8, 9	5	88.3	0	0	2	1.7	2.5	80	0
5, 6	6	42.5	1	2.5	2	7.5	2.5	75	0
2, 3, 4	7	1.7	1	7.7	2	10	2	72.5	0

n.a.: not analyzable, extraosseous localization of the stem; %: mean percentage of osseous integration of matched ROIs; mm: mm of osteolysis.
